# Uterine Natural Killer Cell Heterogeneity: Lessons From Mouse Models

**DOI:** 10.3389/fimmu.2020.00290

**Published:** 2020-02-21

**Authors:** Dorothy K. Sojka

**Affiliations:** Rheumatology Division, Washington University School of Medicine, St. Louis, MO, United States

**Keywords:** conventional NK cells, pregnancy, maternal-fetal interface, tissue-resident NK cells, uterine NK cells

## Abstract

Natural killer (NK) cells are the most abundant lymphocytes at the maternal-fetal interface. Epidemiological data implicate NK cells in human pregnancy outcomes. Discoveries using mouse NK cells have guided subsequent advances in human NK cell biology. However, it remains challenging to identify mouse and human uterine NK (uNK) cell function(s) because of the dynamic changes in the systemic-endocrinological and local uterine structural microenvironments during pregnancy. This review discusses functional similarities and differences between mouse and human NK cells at the maternal-fetal interface.

## Introduction

Concinnity, the harmonious arrangement of different parts skillfully fitting together, is a word that describes the orchestrated changes that occur between mother and conceptus during pregnancy. The coordinated modifications are supported by the maternal immune system which accommodates the genetically distinct individual. There is no direct contact between the circulations of the mother and the conceptus; however, conceptus-derived extraembryonic membranes evolve into the chorioallantoic placenta and the chorioamnion, both directly bordering maternal tissues of the uterine cavity. As these maternal-fetal boundaries are established, uterine immune cells provide support. Survival of mammalian species defies the classic laws of transplantation immunology.

Mammalian reproductive success depends on tightly-regulated signal coordination that lead to the optimal vascular perfusion of a placenta that supports the fetus. In humans, abnormal spiral artery development is associated with obstetrical syndromes such as preeclampsia (1), a hypertensive disorder that affects 3–7% of pregnancies. Epidemiological evidence associates preeclampsia with specific maternal natural killer (NK) cell receptors and their cognate human leukocyte antigen class I (HLA-I) ligands expressed by conceptus cells (2–4). The most abundant maternal leukocytes at the maternal-fetal interface are uterine NK (uNK) cells. Therefore, epidemiological data strongly suggest that uNK cells have crucial roles in remodeling the maternal vessels that support placental development and function over pregnancy.

NK cells express killer Ig-like receptors in human (KIRs) and lectin-like receptors in mouse (Ly49s), which are the primary major histocompatibility complex class I (MHC-I)-specific inhibitory receptors. Pregnant women with a specific KIR haplotype carrying a conceptus with a specific HLA-C genotype have significantly greater risk for preeclampsia (2). Expanded evidence for the negative effects of inhibitory receptors on NK cells interacting with their MHC-I ligands on fetal tissues was obtained from a cohort of African women, who have more genetic diversity in KIR haplotypes and HLA alleles (4). Studies associating specific maternal KIR and conceptus HLA alleles with preeclampsia strongly suggest a precise role for NK cell receptors that recognize fetal MHC-I, although further *in vivo* human studies would be challenging. In mice, specific maternal-fetal MHC-I haplotype combinations display differences in decidual vessels, placental sizes, and fetal weights (5, 6). Thus, studies of murine NK cells can be used to guide future translational investigations of human pregnancy complications despite species-specific differences between the pregnancies (7, 8).

## Maternal-Fetal Interface

Human and mouse pregnancies induce environmental changes in uterus that establish receptivity and implantation (9). The maternal-fetal interface includes maternally-derived decidua basalis and conceptus-derived placenta. In humans, endometrial decidualization occurs each menstrual cycle and is shed if implantation does not take place (10). If implantation occurs the decidual cells proliferate, contact invading extravillous trophoblasts (EVTs) and form the decidua basalis. Unobstructed maternal blood flow into the intervillous space occurs between 10 and 12 weeks, marking the end of the first trimester. In mice, blastocyst implantation triggers decidualization, where uterine stromal cells are transformed into large decidual cells that proliferate to surround the implantation site then become the decidua basalis. In both species, decidualization is accompanied by vascular changes and leukocyte accumulation, largely NK cells (11, 12). At gestational day 9.5, the murine labyrinthine placenta accepts maternal blood flow and supplies nutrients to the fetus for the remainder of the pregnancy. At this time the mesometrial aggregate of pregnancy (MLAp) forms in the maternal uterine wall of mice but not human (11). The definitive chorioallantoic placenta develops, accompanied by vascular remodeling of the subjacent uterine arteries. The maternal-fetal interface is well established by first trimester (humans) and mid-pregnancy (mice).

## Placental Development and Function

A placenta anchors each conceptus to the uterine wall, is the site of nutrient, gas, and waste exchange, and induces an immune environment that nurtures and protects the fetus (13, 14). Placental dysfunction results in human pregnancy complications that associate with long-term health consequences for both mother and baby. The *fetal origins hypothesis*, coined by David J.P. Barker [known as the Developmental Origins of Human Adult Disease (DOHAD)], posits that *in utero* fetal programming mediated by placental function has life-long effects on the baby's health (15, 16). Thus, placenta is pivotal not only to fetal development but also offspring health.

While placental structural anatomy varies, in all species the placenta arises from the trophectoderm of the preimplantation blastocyst (17). This layer is programmed to differentiate into fetal membranes while the inner cell mass of the blastocyst evolves the fetus. The mammalian placenta is categorized based on histological structure of the maternal-fetal interface. There are three placental classifications that includes: epitheliochorial (cow, horse, pig), endotheliochorial (dog, cat), and hemochorial (human, rodent). Hemochorial is the most invasive type, in which fetal trophoblast cells invade deeply into the maternal endometrium and vasculature to establish maternal blood perfusion through the placenta.

### Hemochorial Placenta

Human and mouse hemochorial placentas share many features, but differ in ways that affect immunity. The human placenta is structured as chorionic villous trees that are bathed in maternal blood. A single layer of multinucleated syncytiotrophoblast (SynT) surfaces human placental villous trees and lines the intervillous space perfused by maternal blood. Mononucleated cytotrophoblasts are undifferentiated progenitor cells, subjacent to the SynT, that differentiate and fuse to replenish the SynT. Other cytotrophoblasts differentiate into EVTs, which are located at tips of the anchoring villi and invade the decidua basalis and maternal decidual vessels. During first and into early second trimester, human placental villi are hemodichorial, covered with a continuous bilayer of SynT and cytotrophoblasts. The villous cytotrophoblast layer becomes discontinuous during the second trimester yielding a hemomonochorial structure with only a SynT cell barrier between fetal and maternal blood.

In mouse, the definitive chorioallantoic placenta is labyrinthine with two separate, maze-like vascular systems. Branches of the central arteries traverse the junctional zone then perfuse the fetal labyrinth with maternal blood. The irregularly-shaped junctional zone lies between the decidua basalis and labyrinth. The labyrinth is surfaced by two syncytial layers of trophoblast (SynT I and II) and a mononuclear trophoblast layer adjacent to the maternal blood. The labyrinthine structure comprises the interhemal unit, which continuously thins during gestation. Scanning electron microscopy indicates that midgestational mouse placenta is hemodichorial, with direct maternal blood contact of SynT-I as the cellular trophoblast layer becomes incomplete, exposing areas of SynT-II (18). These differences in cellular composition and placental structure may alter immunity at the maternal-placental interface. Thus, mechanisms that protect fetuses from an activated maternal immune system, and block access of maternally circulating pathogens to the fetus may differ spatiotemporally in human and mouse gestations.

### Immunological Interface

**Figure 1:**
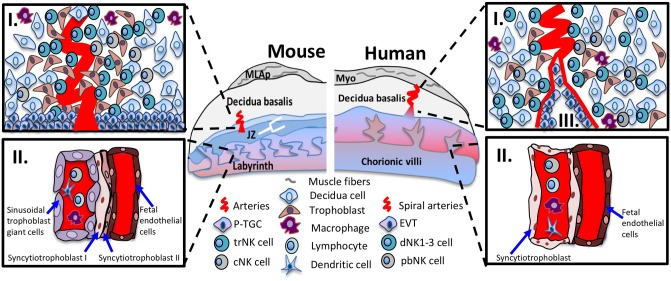
Immunological interfaces of the mouse and human placentas. Schematic diagram of placenta positioned with maternal tissues above fetal tissues. The murine (left panel) and human (right panel) placenta. The top inset **(I)** shows the cellular components of midgestation decidua basalis. In mouse and human decidua basalis, NK cell subsets are in close contact with decidua cells, invading interstitial trophoblast cells and other immune cells such as macrophages and dendritic cells. The bottom inset **(II)** shows the interhemal membrane unit in the placental labyrinth and chorionic villi. The sinusoidal trophoblast giant cells (mouse) and syncytiotrophoblast cells (human) line the maternal blood sinus and are exposed to circulating immune cells among them, cNK cells. In human placentas, the bottom inset **(III)**, shows the endovascular trophoblast invasion where EVTs remodel the maternal vasculature and are exposed to circulating immune cells. Abbreviations in figure: parietal trophoblast giant cells (P-TGCs), junctional zone (JZ), tissue-resident NK cell (trNK), conventional NK cell (cNK), peripheral blood (pNK) cells, decidual NK (dNK) cells, extravillous trophoblasts (EVT).

Typically, placental membranes separate the semi-allogenic fetus from the maternal immune system throughout gestation, an arrangement that protects against maternal, immune-mediated elimination of the fetus. Multiple immunological interfaces occur between the placenta and maternal immune system. One such interface is between the trophoblast cells that line the chorionic villi (human) and labyrinth structure (mouse) that bathe in maternal blood (Figure 1-II). In human placentas, the SynT cells exposed to maternal blood do not express MHC-I or MHC-II, and do not elicit maternal T cell responses. In mouse placentas, the MHC expression on labyrinthine trophoblast is less clear. A second immunological interface occurs in the decidualized endometrium. In a process called interstitial trophoblast invasion the EVTs in human and extra labyrinthine trophoblasts in mice are intimately positioned to interact with the maternal immune cells. For example, EVTs express the class I molecule HLA-C and non-classical class I molecules HLA-E and HLA-G. HLA-C, HLA-E, and HLA-G interact with KIR and CD94/NKG2 receptors expressed in NK cells. HLA-G binds, members of the immunoglobulin-like transcript (ILT) ILT2 and ILT4, a family of receptors expressed in NK cells to induce growth factors important for fetal development (19). Invading giant trophoblast cells (TGCs) in mouse do not express non-classical MHC-I molecules (20, 21); however, TGCs that potentially contact immune cells in the decidua basalis of the C57BL/6J B6 mice do express the classical MHC-I, H-2K (Figure 1-I). Finally, in human placentas during endovascular trophoblast invasion, the EVTs hijack and transform the maternal circulatory system to yield high capacitance, low resistance arterial flow in the placenta and interact with circulating immune cells (Figure 1-III). Hence, the different immunological interfaces, with differential expression of paternally-inherited MHC receptors, may elicit distinct maternal immune responses such as systemic responses to SynT, or local tissue-specific responses to invading trophoblasts, or both.

## NK Cells in Pregnancy

During pregnancy, uNK cells dominate at the implantation site in species with hemochorial placentation (9, 22–24). Approximately 70% of lymphocytes at the maternal-fetal interface are uNK cells during early human and mouse gestation; this percentage declines after mid-pregnancy (25). Histologically, uNK cells are localized to human decidua basalis and mouse junctional zone and MLAp (26, 27). Notably, they are essentially absent from the mouse placental labyrinth (27). Here, “uNK” cells will refer to NK cells in human and mouse implantation sites. Emerging evidence indicates that uNK cells are heterogeneous and contribute to pregnancy success, although they are phenotypically and functionally distinct from conventional NK cells in the circulation (28–31).

### cNK Cells

NK cells are the originating members of an assortment of innate lymphoid cells (ILCs). NK cells are a heterogeneous population in the spleen, circulating blood, and many tissues (27, 32–34). Historically, most studies investigated conventional NK (cNK) in mouse spleen and human peripheral blood. NK cells do not express antigen-specific T-cell receptors (TCRs) or B-cell receptors (BCRs), distinguishing them from the adaptive immune system (35, 36). Developmental studies indicate that all ILC lineages arise from common lymphoid progenitors (CLPs) (37, 38). CLPs differentiate into NK cells, ILC1s, ILC2s, ILC3s and lymphoid tissue inducer (LTi) cells (39, 40). The common helper ILC precursor (CHILP), the progenitor to all helper-like ILC lineages, gives rise to ILC1s, ILC2s, and ILC3s, but not LTi cells or NK cells (41). This separates cNK cell development from other ILCs and ensures that cytotoxic NK cells are distinct from ILC1s (38).

### trNK Cells

**Figure 2:**
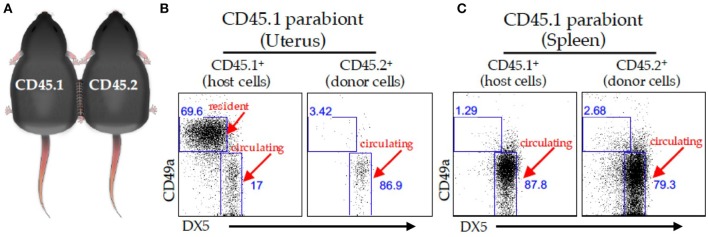
Parabiosis model. **(A)** Schematic of two congenically marked animals that were surgically joined together. C57BL/6J (CD45.2) mice were parabiosed to congenic B6-Ly5.1 (CD45.1) mice. The organs were harvested on day 14 post-parabiosis surgery and analyzed using flow cytometry. A representative dot plot of the virgin uterus **(B)** or spleen **(C)** gated on live CD3^−^ CD4^−^ NK1.1^+^ cells followed by a CD45.1 gate (left panels) and CD45.2 gate (right panels) in the CD45.1 parabiont. The percentages of CD49a^+^ and DX-5^+^ are depicted in the dot plots.

We identified tissue-resident NK (trNK) cell populations in mouse liver, skin, and virgin uterus that are distinct from cNK cells (32). The trNK cells do not circulate in parabiotic mice, whereas cNK cells circulate (Figure 2). Phenotypic and RNA-seq analyses revealed that trNK and cNK cells express CD49a and DX5, respectively (32, 33). Liver trNK cells lack Eomesodermin (Eomes), a transcription factor expressed in cNK cells. *Nfil3*^−/−^ mice have trNK cells in liver, skin, and virgin uterus, but lack cNK cells. *Tbx21*^−/−^ mice lack trNK cells in liver and skin, whereas cNK cell distribution is relatively unaffected at these tissues. In virgin uteri, trNK cells dominate over cNK cells and are unaffected in *Nfil3*^−/−^ and *Tbx21*^−/−^ mice. Ly49 receptor expression repertoires differ between trNK and cNK cells; liver trNK cells do not express Ly49D or Ly49H. Although trNK cells have an immature phenotype, they have more diverse cytokine production than cNK cells. Thus, trNK and cNK cells represent divergent NK cell lineages, with a distinct trNK cell lineage in virgin uterus differing not only from cNK cell but also from liver/skin trNK cells (32).

### Conversion of cNK Cells

Although trNK and cNK cells represent different lineages, recent studies suggest cNK cell conversion to NK cells phenotypically similar to liver ILC1 cells (42). For example, mouse CD49a^−^ DX5^+^ Eomes^+^ cNK cells can change their phenotype in a tumor microenvironment to become CD49a^+^ DX5^−^ Eomes^int^, while, cNK cells convert into ILC1-like cells during *Toxoplasma gondii* infection (43). Human peripheral blood (pb) NK cells and hematopoietic progenitor cells isolated from decidual tissue also convert *in vitro* to a decidual NK cell phenotype (44, 45). It is currently unknown whether cNK cell conversion occurs in pregnancy.

### Origin of uNK Cells

Despite differences in gestational lengths (9 months in humans vs. 19.5 days in C57BL/6J mice) and placental structure, early pregnancy consistently triggers lymphocyte accumulation within successful implantation sites (23, 46). Although uNK cells are ultimately derived from bone marrow (26, 47), studies using immunocompetent donor and alymphoid recipient mice report uNK cell accumulation during pregnancy using uterine segment transplants (48, 49) or adoptive splenocyte transfer (48). These results indicate uterine homing of peripheral NK cells when the uterus lacked endogenous uNK cells. These findings were challenged when adoptively transferred splenic NK cells failed to home to a syngeneic, immunocompetent pregnant uterus, indicating expansion of resident progenitor NK cells (50). We recently used the parabiosis model together with experimentally-induced decidualization to study early pregnancy events and demonstrated that trNK cells do not circulate. Rather, trNK cells proliferate locally and expand the uNK cell pool with minimal cNK cells contributions (13, 27). These data support a two-wave hypothesis for uNK cell accumulation during typical pregnancies (13, 27, 51). The first wave involves local proliferation of trNK cells during decidualization; the second wave postulates the accumulation of cNK cells recruited into the decidua basalis during placentation. Whether or not trNK cells contribute to the NK cell pool of early human pregnancy remains unknown (discussed below).

### Human NK Cells

In first trimester human pregnancy, NK cells represent ~70% of the lymphocytes present in decidua basalis and are phenotypically and functionally distinguished from pbNK cells. Human uNK (often referred to as decidual NK cells, dNK) cells are CD56^bright^ CD16^−^KIR^+^CD9^+^CD49a^+^ while pbNK cells are CD56^dim^CD16^+^KIR^+^ (52). Compared to pbNK cells, uNK cells are not cytotoxic, produce lower amounts of IFN-γ and, as in mouse, secrete VEGF that promotes angiogenesis (28, 53, 54). Single-cell RNA sequencing comparing cells isolated from first trimester decidual basalis and matched peripheral blood demonstrated three distinct NK cell subsets in decidua basalis, dNK1-3, distinct from pbNK cells (55). High dimensional CyTOF analysis found dNK1-3 mixed with populations of proliferating dNK, dILC3 and pbNK cells suggestive of mixed origins of decidual lineages (31). The dNK1 cells resembled a recently identified “pregnancy trained dNK cell” subset found in repeated pregnancy (56) similarly to expanded ILC1s of mouse second pregnancy (57). Mouse models should be instrumental in clarifying the functional contributions of each uNK cell subset at the maternal-fetal interface.

## NK Cell Function in Pregnancy

### Arterial Remodeling

Understanding of how human uNK cells affect pregnancy and the placental vasculature has been challenging; however, studies of mouse uNK cells have been informative. Several mouse genetic models deficient in NK cells display aberrant modification of the uterine gestational vasculature, as their strongest reproductive phenotype. *Rag2*^−/−^*gc*^−/−^ alymphoid mice, display defective spiral artery remodeling during pregnancy (58) but no defects in implantation (59). The arterial defects were rescued in bone marrow (BM) chimeric mice when *Rag2*^−/−^ *gc*^−/−^ recipients received wild-type BM but not BM from interferon-γ-deficient mice. Similarly, *Nfil3*^−/−^ mice that lack cNK cells (32), have a smaller MLAp, aberrant uterine arterial modifications, and smaller pups (60, 61). Roles for interferon-γ in dNK cell-mediated vessel modification have not been confirmed in human studies. In the first trimester, uterine artery Doppler ultrasound screening can be used to identify women with poor spiral arterial remodeling predicting greater risk for preeclampsia and fetal growth restriction (62). Using *in vitro* analysis of EVT lines and primary explant cultures, dNK cells from the typical pregnancies promoted trophoblast motility and invasion while dNK cells from women identified as high risk for preeclampsia limited trophoblast function (63). Taken together, human and murine placental pathology supports that vascular remodeling defects are associated with preeclampsia and fetal growth restriction and linked to deficits in uNK cells numbers or functions (64, 65).

### Border Control at the Maternal-Fetal Interface

Establishing hemochorial placentation requires aggressive invasion by trophoblast cells. Human trophoblasts show interstitial invasion, penetrating the uterine epithelium and expanding in the subjacent decidua. Rodent trophoblast invasion is significant but not as deep as human (66). Modifications of maternal blood flow are established by endovascular trophoblast invasion of implantation site decidual vessels. In humans and rats, interstitial and endovascular trophoblast invasion both extend past the decidua basalis and penetrate the inner myometrium. In mice, trophoblast invasion is interstitial and limited to the junctional zone (67). Uncontrolled trophoblast invasion that penetrates beyond the decidua into the myometrial muscle creates the life-threatening pregnancy complication called placenta accreta. A recently reported dystocia mouse model resembles human placenta accreta (60). The phenotype includes stillbirths, hemorrhage, and undelivered placentas. Histopathology revealed excessive trophoblast invasion and reduced numbers of trNK cells (68). In this model, a deficiency in the signaling adaptor molecule Grb2-associated binding protein 3 (Gab3) was important for trNK cell proliferation in response to local cytokine (IL15) stimulation. A similar outcome of dysregulated trophoblast invasion occurs in a rat model of IL15 deficient NK cells (69). These results suggest that defects in trNK cell proliferation lead to interstitial trophoblast hyperinvasion and provide insights into placenta accreta.

## Mouse Models

The mouse is a well-characterized genetic model system to study NK cell biology because defined mice allow reproducible experimental conditions. Discoveries in mouse NK cell biology guided subsequent studies on human NK cells. For example, Ly49A was identified as an inhibitory MHC-I–specific NK cell receptor (70) before molecular identification of KIRs (71, 72). Liver trNK cells were identified in mouse (32, 33) prior to human liver trNK cell discovery (73). Endometrial sampling of ongoing human gestation is extremely limited to the narrow window for elective pregnancy termination. Sampling at delivery is more accessible, but uNK cells are rare or absent by term in both women (74, 75) and mice (76), rendering term tissue samples inappropriate for defining normal uNK cell functions. Therefore, animal models are crucial for insights into uNK cell biology as complements to the sparse data available from human implantation sites.

## Conclusion

Integration of data derived from experimental animal models with the limited data available from human implantation sites is essential to advance our knowledge of uNK cell biology. Despite their name, uNK cells are not good killers in pregnancy but promote placentation in response to allogeneic MHC molecules expressed on human extravillous, or mouse extra-labyrinthine trophoblasts. Importantly, the maternal immune system in pregnancy does not defy the classical laws of transplantation, but actually follows these laws, in a unique and highly modified format.

## Author Contributions

The author confirms being the sole contributor of this work and has approved it for publication.

### Conflict of Interest

The author declares that the research was conducted in the absence of any commercial or financial relationships that could be construed as a potential conflict of interest.
